# A Bioinformatics Filtering Strategy for Identifying Radiation Response Biomarker Candidates

**DOI:** 10.1371/journal.pone.0038870

**Published:** 2012-06-29

**Authors:** Jung Hun Oh, Harry P. Wong, Xiaowei Wang, Joseph O. Deasy

**Affiliations:** 1 Department of Medical Physics, Memorial Sloan-Kettering Cancer Center, New York, New York, United States of America; 2 Department of Infectious Diseases, Washington University School of Medicine, St. Louis, Missouri, United States of America; 3 Department of Radiation Oncology, Washington University School of Medicine, St. Louis, Missouri, United States of America; National Institutes of Health, United States of America

## Abstract

The number of biomarker candidates is often much larger than the number of clinical patient data points available, which motivates the use of a rational candidate variable filtering methodology. The goal of this paper is to apply such a bioinformatics filtering process to isolate a modest number (<10) of key interacting genes and their associated single nucleotide polymorphisms involved in radiation response, and to ultimately serve as a basis for using clinical datasets to identify new biomarkers. In step 1, we surveyed the literature on genetic and protein correlates to radiation response, *in vivo* or *in vitro*, across cellular, animal, and human studies. In step 2, we analyzed two publicly available microarray datasets and identified genes in which mRNA expression changed in response to radiation. Combining results from Step 1 and Step 2, we identified 20 genes that were common to all three sources. As a final step, a curated database of protein interactions was used to generate the most statistically reliable protein interaction network among any subset of the 20 genes resulting from Steps 1 and 2, resulting in identification of a small, tightly interacting network with 7 out of 20 input genes. We further ranked the genes in terms of likely importance, based on their location within the network using a graph-based scoring function. The resulting core interacting network provides an attractive set of genes likely to be important to radiation response.

## Introduction

In the ‘omics’ era, the number of biomarker candidates potentially available for statistical testing is often much larger than the number of patient data points. This presents a fundamental problem in biomarker research: the number of candidate genetic or epigenetic markers often overwhelms the inherent statistical power available in a clinical dataset, which usually has tens or hundreds of patient cases available rather than thousands. This statistical mismatch is typically becoming worse as more of the intracellular complexity of molecular machinery is identified. At one extreme, a genome-wide association study (GWAS) examining the correlations of millions of tag single-nucleotide polymorphisms (SNPs) to cancer treatment outcome may require a very high, and biologically unlikely, odds ratio given the number of multiple comparisons, to reach statistical significance. At the other extreme, it is clear that investigators cannot a priori identify the most important biomarker genes or SNPs for testing. These unsatisfying extreme cases motivated our search for a middle strategy that would objectively identify a modest number of promising SNPs/proteins, etc. as a cohort for testing against a given dataset. Because clinical datasets for a given endpoint are commonly of modest size (tens or hundreds, not thousands, of patients), we searched for key protein interaction networks that result in less than approximately a hundred candidate SNPs. Our methodology, of course, could be adopted to throw a wider net if much larger datasets become available. Our endpoint of interest is late toxicity following radiation therapy for cancer. Many cancer patients who receive radiation therapy suffer from acute or late side effects; the risk for experiencing these side effects is expected to have a genetic component [1]. Numerous genes participate in a cascade of events in response to radiation and the resulting DNA damage in a complex signal transduction network [2].

Recently, many studies have focused on finding radio-responsive genes at the whole genome level with gene expression microarrays. Rieger and Chu used oligonucleotide microarrays to develop a genome-wide portrait of transcriptional response to ionizing radiation (IR) and ultraviolet (UV) radiation in cell lines collected from 15 healthy individuals [3]. In another study [1] using samples extracted from cancer patients with acute radiation toxicity, Rieger *et al.* showed that toxicity after radiation therapy (radiotherapy) could be associated with abnormal transcriptional responses to DNA. Jen and Cheung [2] assessed transcriptional levels of genes in lymphoblastoid cells at various time points with 3 Gy and 10 Gy of *ex vivo* IR exposure. Following 10 Gy of IR exposure, more genes were induced, suggesting that a higher radiation dose causes a more complex response. A high percentage of significant genes were involved in cell cycle, cell death, DNA repair, DNA metabolism, and RNA processing. Eschrich *et al.* [4] analyzed microarray gene expression data derived from 48 human cancer cell lines and generated an interaction network using MetaCore software (GeneGo, Encinitas, CA) with the top 500 genes identified by linear regression analysis. Subsequently, based on 10 hub genes obtained from the network, they modeled radiosensitivity (survival fraction at 2 Gy) using a linear regression method.

Normal tissue toxicity after radiotherapy may partially be attributable to specific genetic mutations. In an effort to identify candidate polymorphisms at the SNP level involved in the cellular response to irradiation in breast and prostate cancers, Popanda *et al.* [5] surveyed many published studies that show associations of SNPs in candidate genes with acute or late side effects of radiotherapy. Andreassen and Alsner [6] summarized studies published on genetic variation in normal tissue toxicity and proposed a model of allelic architecture that illustrates relative risk for genetic variants associated with normal tissue radiosensitivity.

In this study, we attempted to define an objective method for identifying key radiosensitivity genes likely to have a significant impact on clinical outcome following radiotherapy. We elected to construct a staged filter. The first step was a comprehensive literature review of radiosensitivity-related genes. These genes were then further delimited to genes responding to IR in an analysis of publicly available microarray gene expression datasets. We further focused the search on interacting networks, based on the hypothesis that good biomarkers are likely to be embedded in important pathways or networks involving multiple genes known to be important to the endpoint in question [7]. This last step may potentially add new, previously unreported targets, based on curated pathway libraries.

## Materials and Methods

In summary, we used a multi-component filtering process: (1) genes associated with radiation response in the literature and (2) genes associated with radiation response in two microarray mRNA datasets. Overlapping genes from these three sources were fed into a curated protein interaction network system (MetaCore) to identify key interacting networks. The most important network was taken as our target set.

### Literature Review of Radiosensitivity-related Genes

We attempted a complete literature review of all genes implicated in radiation response. Published papers were searched by using PubMed and Scopus search engines in 2010 and by following citations within the identified papers. The search strategy was based on a combination of the following search keywords: “SNPs, polymorphisms, or microsatellites” and “irradiation, radiation, or radiotherapy” and “morbidity, radiosensitivity, normal tissue, toxicity, or complications” and “siRNA, knockdown, or knockout”. Papers referred to in the original search returns, or referring to the original papers at a later date were also reviewed. This resulted in an in-depth review of around 200 published papers, and a list of 221 genes implicated in radiation response.

### Microarray Gene Expression Datasets

To identify significant radio-responsive genes based on microarray gene expression profiling, we searched for all relevant, publically available microarray datasets, resulting in locating two datasets. We analyzed GSE1977 and GSE23393, downloaded from the publicly available Gene Expression Omnibus (GEO) database (http://www.ncbi.nlm.nih.gov/geo/). In GSE1977, lymphoblastoid cell lines obtained from 15 healthy individuals were established by immortalization of peripheral blood B-lymphocytes [3]. The response of numerous genes was measured by mock treatment, UV, and X-ray exposures. Cells were exposed to 5 Gy radiation doses and harvested for RNA 4 hours later. In our work, the differential between mock and X-ray cases was used. In contrast, in GSE23393 [8], blood was gathered from eight radiotherapy patients (at our institution): eight samples were collected immediately before irradiation and another eight samples were collected at 4 hours after total body irradiation with 1.25 Gy X-rays.

### Preprocessing for Identification of Significant Genes

Before the microarray datasets were analyzed, gene expression values were log-base-2 transformed, followed by quantile normalization across all samples [9]. Microarray gene expression values from two different conditions (before and after exposure) were compared using a two-tailed t-test to identify differentially expressed genes (radio-responsive genes). To estimate the likelihood of identifying significant genes by chance, we computed permutation-based *p*-values using 10,000 permutations. Then, using Storey’s method, the false discovery rate (FDR) and *q*-value for each gene were calculated [10]. Significance Analysis of Microarrays (SAM) and t-test are widely used for indentifying differentially expressed genes in the analysis of microarray data [11]. We chose a permutation t-test with an assumption that the permutation t-test and SAM could yield a set of similar significant genes, as recommended by Chen *et al*. [11]. In this analysis, we did not use a fold change cutoff in order to avoid losing some important genes, a problem described by Larsson *et al*. [12].

### Pathway and Process Analysis

Significant genes were identified both in the literature review and the analysis of two microarray datasets (GSE1977 and GSE23393). These genes were then entered into a manually curated pathway analysis database (MetaCore™, GeneGo, Inc., Carlsbad, California). The commercial pathway analysis system, MetaCore, computes *p*-values for overrepresented pathways and processes. MetaCore is based on a comprehensive manually curated attempt to capture protein interactions as networks. We used MetaCore to attempt to find the most probable interaction pathways among a set of genes uploaded by the user. Several algorithms are available to do this; we used the “Analyze network” option. If necessary, MetaCore adds appropriate genes to complete a network.

### Gene Ontology Analysis

A further analysis of the resulting significant genes was performed using the Gene Ontology (GO) database (www.geneontology.org), in which genes are annotated with known molecular functions, biological processes, and cellular component locations.

### Gene Ranking

In our previous work to identify blood-based protein biomarkers to predict radiation-induced pneumonitis [7], we proposed a graph-based scoring function to rank proteins in a protein–protein interaction network. The network consisted of candidate proteins we identified in mass spectrometry analysis and four previously identified (‘regularization’) biomarker proteins. Using the proposed method, we attempted to measure a ‘functional distance’ between each candidate protein and the four regularization proteins, based on the hypothesis that some proteins relevant to a specific disease exist in close proximity, in a network sense. In the current study, we modified that algorithm such that within a given protein–protein interaction network for a biological process, we estimate the functional distance between each protein and all the remaining proteins in the network, since all the proteins in the network are more likely to be related to one another and act together in the biological process.

To rank biomarkers, we modelled each protein–protein interaction network as a directed graph, G = (V, E), where V consists of a set of nodes (proteins) and E is the set of possible edges (protein–protein interactions) between pairs of nodes. Let *A* and *B* be two proteins in a network. We assume that there are two concepts of distance between *A* and *B*: a geometrical distance that is defined in terms of the number of nodes in the shortest path between *A* and *B*, as well as a virtual distance that is defined in terms of the number of publications that verify the interactions along the shortest path. Intuitively, as the number of intermediate nodes between *A* and *B* increases, the geometrical distance increases and the two proteins are less likely to be correlated. In contrast, considering virtual distance, we expect that as the number of references demonstrating a relationship between two proteins increases, they are more likely to be related. In other words, the number of references is proportional to relatedness while the number of nodes is inversely proportional.

Using a power law, we calculate two scores from *A* to *B*: a reference score (*rs*) and a node score (*ns*) as follows:
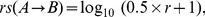
(1)

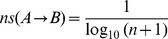
(2)where *r* and *n* are the total number of references and nodes in the shortest path from *A* to *B*. We suppose that the influence of the number of nodes is greater than that of the number of references. Therefore, as the number of intermediate nodes between any two given nodes increases, the relationship between the two nodes becomes much less likely. The score capturing the path from *A* to *B* is defined as the summation of two different scores:
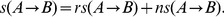
(3)Likewise, we also estimate a score from B to A, 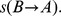 Then, the final score, 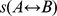 between A and B, is defined as the maximal value among 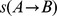 and 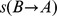:



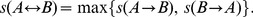
(4)We suppose that the final score of a protein is computed by the summation of all scores between the protein and all the remaining proteins in the network. Hence, the final score of a protein *A* is defined by:
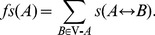
(5)


To estimate the number of references and nodes, we employed two methods. For the number of references, we used a function in the MetaCore software that provides the number of references between two connected proteins in a network. For the number of nodes, we used the Floyd-Warshall algorithm that was originally designed to find the shortest paths between all pairs of nodes based on dynamic programming [13]. To apply this algorithm to our problem of estimating the number of nodes, we modified the original Floyd-Warshall algorithm such that an equal weight of 1 was assigned to all connected edges in a network. As a result, the modified algorithm generated a matrix that represents the number of nodes on the all-pairs shortest-paths in a given protein–protein interaction network.

## Results

### Identification of Significant Biomarkers via Literature Review

Based on the literature review, several types of biomarkers, including genes, proteins, kinases, ligands, and protein complexes were identified. To unify the biomarker terms differently used across studies, we converted all the biomarkers into their corresponding gene symbols. As a result, 221 unique genes and 4 protein complexes (DNA-PK, HSP70, MRN(95), RAS) were identified from around 200 papers that studied radiation response-related biomarkers [4], [14]–[185]. **Table 1** displays the 221 unique genes and their corresponding GO processes, including DNA repair, cell proliferation/cycle, apoptosis, RNA processing, and response to stress. It is well known that ionizing radiation causes DNA damage that activates the p53 pathway through ATM [186]. Genes that are involved in cell cycle, such as CDKN1A, GADD45A, MDM2, and CCNG1, are known to be dependent on p53 [2]. Also, other cell cycle-related genes including CCNB1 and CDC20 were identified. Among cell cycle or proliferation genes, TOB1, BTG2, and CDKN1A are anti-proliferative/check-point related [3]. Several genes (XPC, DDB2, PCNA, ERCC4, and NBN) are involved in DNA repair. Two major pathways to repair IR-induced DNA double-strand breaks are homologous recombination (HR; genes include XRCC2, XRCC3, MRE11A, RAD50, NBN, BRCA1, and BRCA2) and non-homologous end joining (NHEJ; genes include LIG4, XRCC4, XRCC5, XRCC6, and DNA-PK) [3]. Some genes, including FAS, BBC3, and TNF, are involved in apoptosis [187]. BCL2 and DDR1 are anti-apoptotic.

**Table 1:** Radio-responsive biomarkers identified by literature review and their biological processes.

Gene Symbol	Entrez Gene ID		DNA repair	Cell proliferation		Cell cycle	Apoptosis	Response to stress	Reference
ABCA1	19								[14]
ABL1	25		v		v		v	v	[4]
ACTA2	59								[15]
AEN	64782						v	v	[16]
AKR1B1	231							v	[17]
AKT1	207				v		v	v	[18]
ALAD	210								[19], [20]
ANXA1	301			v	v		v	v	[21]
APEX1	328		v					v	[22]–[26]
APOE	348			v			v	v	[27]
AR	367			v					[4]
ATF3	467			v					[28]
ATM	472		v	v	v		v	v	[25], [26], [29]–[43]
BAD	572			v			v		[44]
BAK1	578						v		[45]
BAX	581						v		[37], [44]–[47]
BAZ1B	9031		v					v	[17]
BBC3	27113						v		[16], [48]–[50]
BCL2	596			v	v		v	v	[37], [44], [46], [51], [52]
BCL2L1	598			v			v	v	[51]
BIRC5	332				v		v		[53]
BRCA1	672		v	v	v		v	v	[54]–[59]
BRCA2	675		v	v	v		v	v	[54]–[57], [59], [60]
BTG2	7832		v	v			v	v	[61]
CAT	847						v	v	[62]
CAV1	857			v				v	[63]
CCNB1	891			v	v			v	[48]
CCND1	595			v	v			v	[46]
CCNE1	898				v				[61]
CCNG1	900				v		v	v	[49]
CD24	100133941			v			v	v	[21]
CD40	958			v				v	[64]
CD68	968								[20]
CD69	969								[16]
CD70	970			v			v		[65]
CD83	9308							v	[21]
CDC20	991			v	v				[66]
CDC6	990			v	v				[61]
CDH2	1000								[21]
CDK1	983			v	v		v	v	[4], [61]
CDK2	1017			v	v				[61]
CDKN1A	1026			v	v		v	v	[15], [16], [37], [47]–[50], [61], [67], [68]
CDKN2A	1029			v	v		v	v	[69]
CHEK1	1111	v		v	v			v	[70], [71]
CLIC1	1192								[72]
CRYAB	1410						v	v	[21]
CSNK2A2	1459				v				[73]
CXCR4	7852						v	v	[15]
CYP2D6	1565								[74]
DCN	1634							v	[21]
DDB2	1643	v						v	[15], [21]
DDR1	780								[15]
DDR2	4921			v					[15]
DDX17	10521								[17]
DRAP1	10589								[17]
DUSP8	1850								[16]
EGFR	1956			v	v		v	v	[46]
EGR1	1958			v					[16]
EGR4	1961			v					[16]
EI24	9538						v		[47]
EIF2AK3	9451						v	v	[75]
EPDR1	54749								[20]
ERBB2	2064			v			v	v	[46], [76]–[78]
ERCC1	2067	v						v	[52]
ERCC2	2068	v		v	v		v	v	[36], [74]
ERCC4	2072	v			v			v	[52], [79]
ERCC5	2073	v					v	v	[52]
FAS	355						v		[47], [80]
FASLG	356			v			v	v	[80]
FDXR	2232								[49], [50], [68]
FGF1	2246			v				v	[81]
FGF2	2247			v	v		v	v	[81]
GADD45A	1647	v			v		v	v	[15], [16], [48], [49], [61], [67]
GBP1	2633								[21]
GDF15	9518								[15], [82]
GFER	2671			v					[83]
GRAP	10750								[16]
GSTA1	2938								[62]
GSTM1	2944								[62], [84]
GSTP1	2950						v		[36], [62], [85], [86]
GSTT1	2952								[62], [84]
H2AFX	3014	v			v			v	[41], [87], [88]
HDAC1	3065			v	v		v		[4], [89]
HERC2	8924	v			v			v	[90]
HSP90AB1	3326							v	[91]
HSP90B1	7184						v	v	[82]
HSPB1	3315						v	v	[91], [92]
HUS1	3364	v			v			v	[93]
ICAM2	3384								[94]
ID3	3399				v		v	v	[20]
IER5	51278								[95]
IFNG	3458			v	v		v	v	[16]
IGF1R	3480			v			v		[46], [96]
IGFBP3	3486			v			v		[21]
IL12RB2	3595			v					[14]
IL17A	3605						v	v	[97]
ILK	3611			v	v		v		[98]
IRF1	3659								[4]
JUN	3725			v	v		v	v	[4], [16]
KRAS	3845								[99]
LIG1	3978	v			v			v	[20]
LIG3	3980	v			v			v	[19], [20]
LIG4	3981	v		v	v		v	v	[35], [60], [74], [100]
LOX	4015							v	[21]
LSM7	51690								[17]
MAD2L2	10459				v				[19]
MAP3K7	6885						v	v	[19], [20]
MC1R	4157	v			v			v	[101]
MCL1	4170						v		[102]
MCM2	4171				v				[61]
MDC1	9656	v			v			v	[35]
MDM2	4193			v	v			v	[16], [49], [52], [103]
MGMT	4255	v					v	v	[20]
MLH1	4292	v			v		v	v	[46], [74]
MMP2	4313							v	[104]
MMP9	4318						v		[105]
MPO	4353						v	v	[62], [106]
MR1	3140								[15]
MRE11A	4361	v			v			v	[29]
MRPL23	6150								[17]
MSH2	4436	v			v		v	v	[46]
MTHFR	4524								[107]
MTOR	2475			v				v	[45]
MYC	4609			v	v		v		[108]
NBN	4683	v		v	v			v	[29], [109]
NEIL1	79661	v						v	[110]
NEK2	4751				v				[111]
NFKB1	4790						v	v	[112]
NNMT	4837								[91]
NONO	4841	v						v	[113]
NOS3	4846			v			v	v	[62], [106]
NOX4	50507			v				v	[114]
NUDT1	4521	v						v	[17]
OGG1	4968	v						v	[115]
PAH	5053								[20]
PAK6	56924								[116]
PARP1	142	v						v	[70], [117]
PCNA	5111	v		v				v	[16], [118]
PER3	8863								[20]
PHLPP2	23035								[119]
PHPT1	29085								[50]
PIK3CA	5290						v		[120]
PIM2	11040			v	v		v		[52]
PLK2	10769				v				[16]
PLK3	1263								[61]
PMS2	5395	v			v			v	[46]
POLB	5423	v					v	v	[121]
POLQ	10721	v						v	[60]
PPA1	5464								[111], [119]
PPM1D	8493			v	v				[15], [122]
PRDX1	5052			v			v	v	[116]
PRDX4	10549								[123]
PRKCB	5579						v		[4]
PRKCZ	5590			v			v		[52]
PRKDC	5591	v					v	v	[124]–[126]
PROCR	10544							v	[21]
PROM1	8842								[127]
PSMB4	5692				v			v	[17]
PSMD1	5707				v				[17]
PTCH1	5727			v					[128]
PTEN	5728			v	v		v		[129]
PTGS2	5743			v	v		v	v	[46]
PTTG1	9232	v			v			v	[19], [130]
RAD21	5885	v			v		v	v	[30], [31], [43], [131]
RAD23B	5887	v						v	[17]
RAD50	10111	v			v			v	[29], [132]
RAD51	5888	v			v			v	[133]
RAD54L	8438	v			v			v	[134]
RAD9A	5883	v			v		v	v	[19], [52]
RALBP1	10928								[135], [136]
RELA	5970			v			v	v	[4], [112]
RND1	27289								[16]
RRM2	6241								[137]
RRM2B	50484	v					v	v	[138]
S100A11	6282			v					[15]
SAG	6295								[139]
SART1	9092				v		v		[20]
SEC22B	9554								[17]
SEPHS1	22929								[140]
SERPINA3	12							v	[20]
SERPINE1	5054			v			v	v	[141]
SESN1	27244			v	v			v	[49], [50]
SIRT1	23411	v		v			v	v	[142]
SMPD1	6609						v		[81]
SOD1	6647	v					v	v	[143]
SOD2	6648			v			v	v	[25], [26], [30], [31], [36], [62], [144], [145]
SRC	6714								[146]
SRF	6722							v	[17]
STAT1	6772			v			v	v	[4], [147]
STAT3	6774							v	[148], [149]
SUMO1	7341	v						v	[4]
TGFB1	7040			v	v		v	v	[25], [26], [30], [36], [43], [145], [150]–[154]
TNF	7124			v	v		v	v	[155]
TNFRSF10B	8795						v		[47]
TNFRSF1A	7132						v	v	[112]
TNFSF10	8743						v		[156]
TNFSF9	8744			v			v		[16]
TOB1	10140			v					[157]
TOP2A	7153	v					v	v	[158]
TOR1AIP1	26092								[16]
TP53	7157	v		v	v		v	v	[37], [41], [46], [48]
TP63	8626			v	v		v	v	[159]
TPP2	7174								[160]
TRAF2	7186						v	v	[161]
TRAF4	9618						v	v	[16]
TXN	7295			v					[162]
TXNRD1	7296			v					[163]
UBB	7314				v		v		[17]
UHRF1	29128	v		v	v			v	[164]
UIMC1	51720	v			v			v	[165]
VEGFA	7422			v			v	v	[46], [166]
WRN	7486	v					v	v	[110]
WT1	7490			v			v		[167]
XIAP	331						v	v	[49], [168], [169]
XPC	7508	v			v			v	[124], [170]
XRCC1	7515	v						v	[22,23,25,26,30,31,36,43,115,
									144,145,150,154,171–175]
XRCC2	7516	v			v		v	v	[109]
XRCC3	7517	v						v	[25,26,30,31,74,109,115,134,
									144,145,172,174–176]
XRCC4	7518	v		v			v	v	[164]
XRCC5	7520	v		v			v	v	[52], [60], [172], [177]–[179]
XRCC6	2547	v						v	[20], [176], [178], [180], [181]
DNA-PK									[52], [182]
HSP70									[183]
MRN(95)									[184]
RAS									[185]

**Figure 1:**
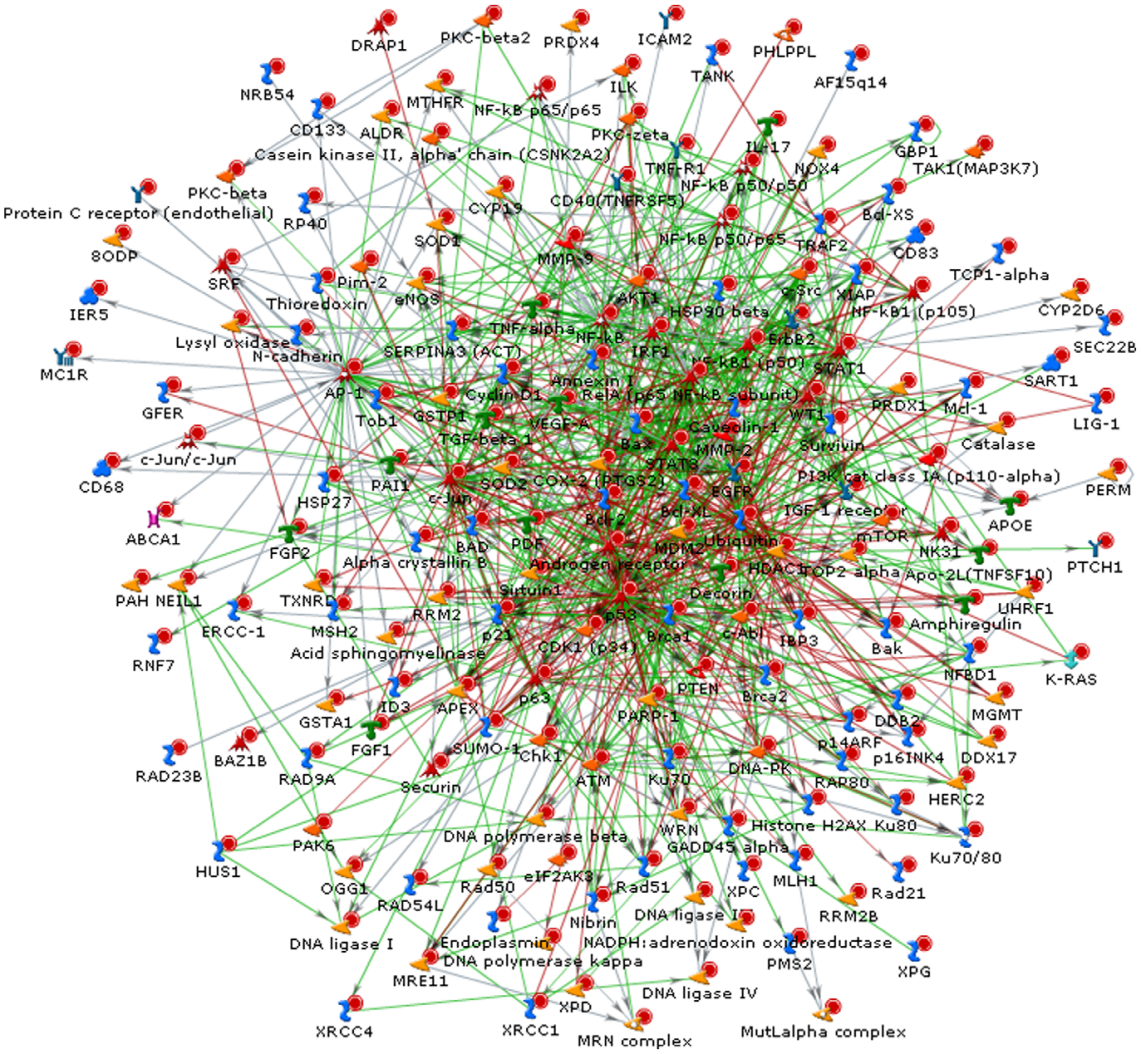
Direct protein-protein interaction network. A network representation that illustrates the complexity of direct connections among genes identified via literature review.

For biological process and pathway analysis, the 221 unique genes were uploaded into the MetaCore. **Figure 1** illustrates a direct interaction network generated with these genes. As shown, numerous genes are strongly connected to one another, suggesting that interacting genes are more likely to play related roles. **Table 2** shows the top ten GeneGo pathways, GeneGo processes, and GO processes. As can be seen in the table, the most highly ranked pathways and processes are associated with DNA damage and repair, cell cycle, and apoptosis.

**Table 2:** The top ten GeneGo pathways/processes and GO processes resulting from genes identified via literature review.

Ranking	GeneGo Pathways
1	DNA damage_Role of Brca1 and Brca2 in DNA repair
2	DNA damage_ATM/ATR regulation of G1/S checkpoint
3	DNA damage_NHEJ mechanisms of DSBs repair
4	DNA damage_Brca1 as a transcription regulator
5	Signal transduction_AKT signaling
6	Some pathways of EMT in cancer cells
7	Apoptosis and survival_Ceramides signaling pathway
8	Signal transduction_PTEN pathway
9	Transcription_P53 signaling pathway
10	DNA damage_ATM/ATR regulation of G2/M checkpoint
**Ranking**	**GeneGo Processes**
1	DNA damage_Checkpoint
2	DNA damage_DBS repair
3	Cell cycle_G1-S Growth factor regulation
4	DNA damage_BER-NER repair
5	Cell cycle_Meiosis
6	DNA damage_Core
7	Apoptosis_Apoptotic nucleus
8	Cell cycle_G1-S Interleukin regulation
9	Development_EMT_Regulation of epithelial-to-mesenchymal transition
10	Cell cycle_S phase
**Ranking**	**GO Processes**
1	Cellular response to stimulus
2	Cellular response to stress
3	Response to stress
4	Regulation of programmed cell death
5	Regulation of cell death
6	Regulation of apoptosis
7	Response to DNA damage stimulus
8	Response to stimulus
9	DNA repair
10	Response to organic substance

### Identification of Significant Genes via Microarray Dataset Analysis

**Figure 2:**
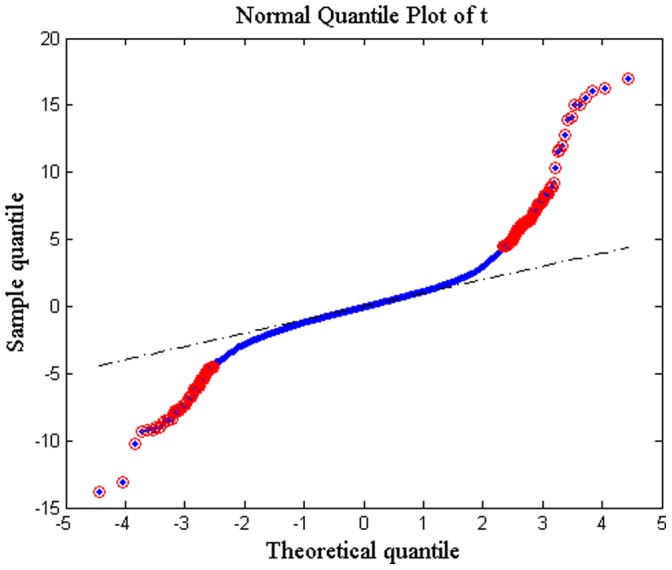
A normal quantile plot of t-scores for GSE1977. Significant genes have red circles.

**Figure 3:**
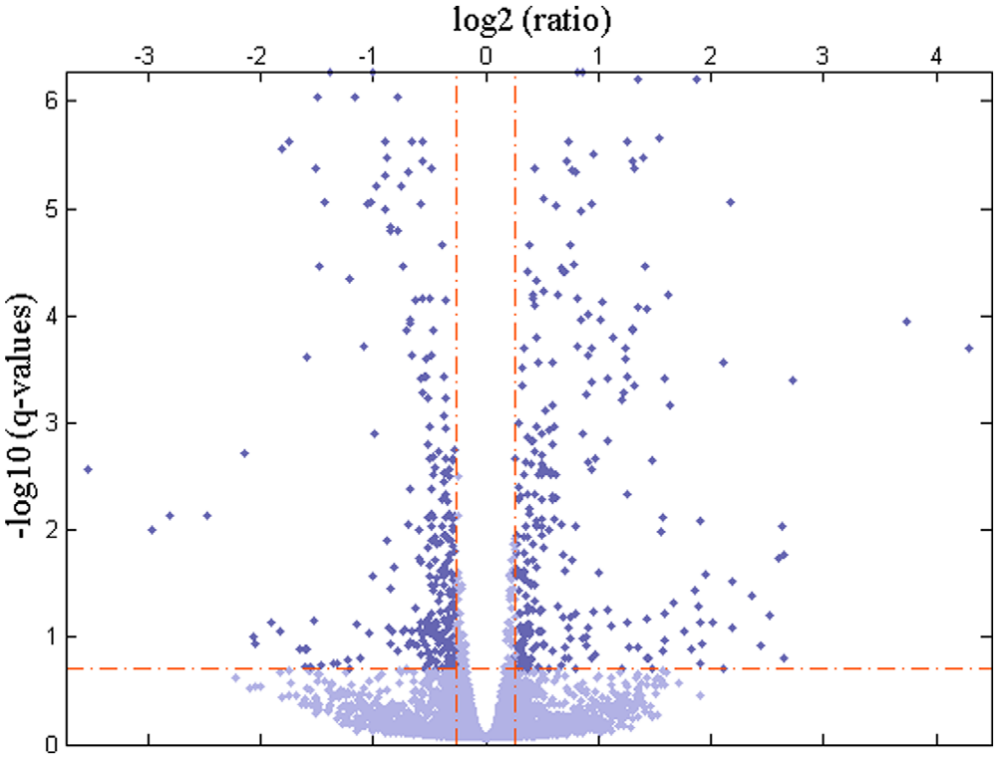
Significant gene detection. A volcano plot that depicts the –log10 of *q*-values against log2 of fold changes for all genes in GSE1977.

To identify significant changes in gene expression values between the two groups (before and after irradiation) in two microarray datasets, a t-test with 10,000 permutations was performed. To estimate p-values, we counted the number of permutations for each gene whose t-scores are greater than or equal to the t-score calculated with observed values. Then, the number of permutations passed the criterion was divided by the total number of permutations [188]. With an FDR of 20%, 631 probes (corresponding to 550 unique genes) were significantly identified for GSE1977. **Figure 2** shows a normal quantile plot of t-scores for GSE1977. Data points of genes that are farther away from the black diagonal line are considered to be differentially expressed. **Figure 3** displays a volcano plot that depicts the –log10 of *q*-values against log2 of fold changes for all genes. The majority of genes with an FDR of 20% changed 1.2-fold or higher. For GSE23393, with an FDR of 20%, 224 probes (corresponding to 184 unique genes) were identified (**Figure S1** and **Figure S2**).

### Overlapping Genes

**Figure 4:**
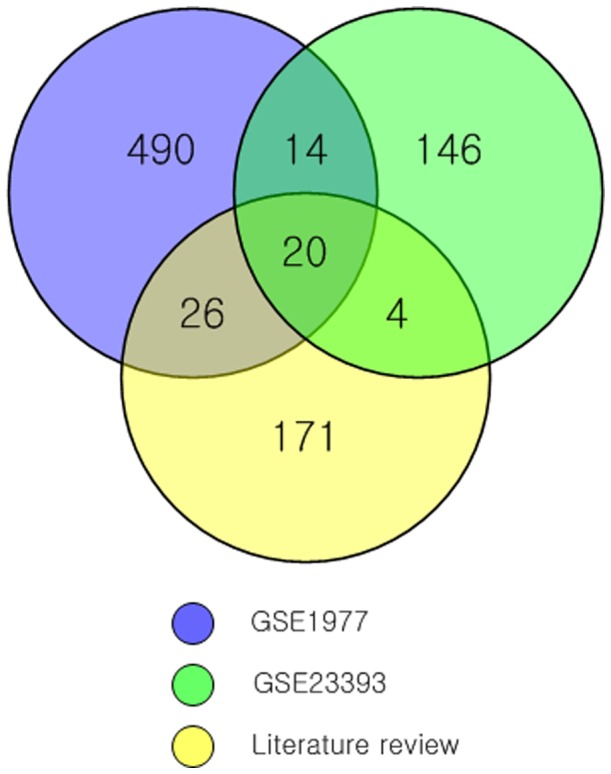
Comparison of significant genes among three sources. A Venn diagram depicting the number of shared and unique genes among a set of genes identified by literature review and two sets of genes identified in the analysis of two gene microarray datasets.

To delimit our potential biomarker set, we investigated which genes are commonly or uniquely found among the set of genes identified by our literature review and two sets of genes identified in the analysis of the two gene microarray datasets, as summarized in **Figure 4** **.** The rationale is that those are genes likely to be key to an active response, but unlikely to be false positives due to the literature review. Twenty genes were commonly identified among the three different analyses (literature review and two microarray datasets), as shown in **Table 3** **.** We further analyzed pathways and biological processes associated with the 20 genes. **Table 4** shows the top ten GeneGo pathways generated by the MetaCore software (**Table S1**). Not surprisingly, even with the 20 genes, DNA damage/repair and apoptosis-related pathways were highly ranked.

**Table 3:** Twenty genes commonly identified by literature review and analysis of two microarray datasets.

Gene Symbol	Entrez ID	Gene Name
ACTA2	59	actin, alpha 2, smooth muscle, aorta
BAX	581	BCL2-associated X protein
BBC3	27113	BCL2 binding component 3
BTG2	7832	BTG family, member 2
CCNG1	900	cyclin G1
CD70	970	CD70 molecule
CDKN1A	1026	cyclin-dependent kinase inhibitor 1A (p21, Cip1)
DDB2	1643	damage-specific DNA binding protein 2, 48 kDa
EI24	9538	etoposide induced 2.4 mRNA
FDXR	2232	ferredoxin reductase
GADD45A	1647	growth arrest and DNA-damage-inducible, alpha
MDM2	4193	Mdm2 p53 binding protein homolog (mouse)
MR1	3140	major histocompatibility complex, class I-related
MYC	4609	v-myc myelocytomatosis viral oncogene homolog (avian)
PCNA	5111	proliferating cell nuclear antigen
PLK2	10769	polo-like kinase 2
PLK3	1263	polo-like kinase 3
PPM1D	8493	protein phosphatase, Mg2+/Mn2+ dependent, 1D
TNFRSF10B	8795	tumor necrosis factor receptor superfamily, member 10b
XPC	7508	xeroderma pigmentosum, complementation group C

**Table 4:** The top ten GeneGo pathways generated by MetaCore when the 20 overlapping genes were used.

#	GeneGo Pathways
1	DNA damage_Brca1 as a transcription regulator
2	DNA damage_ATM/ATR regulation of G1/S checkpoint
3	Signal transduction_AKT signaling
4	Apoptosis and survival_Apoptotic TNF-family pathway
5	DNA damage_ATM/ATR regulation of G2/M checkpoint
6	Apoptosis and survival_p53-dependent apoptosis
7	DNA damage_Role of Brca1 and Brca2 in DNA repair
8	DNA damage_Nucleotide excision repair
9	Transcription_P53 signaling pathway
10	Cytoskeleton remodeling_TGF, WNT and cytoskeletal remodeling

**Figure 5:**
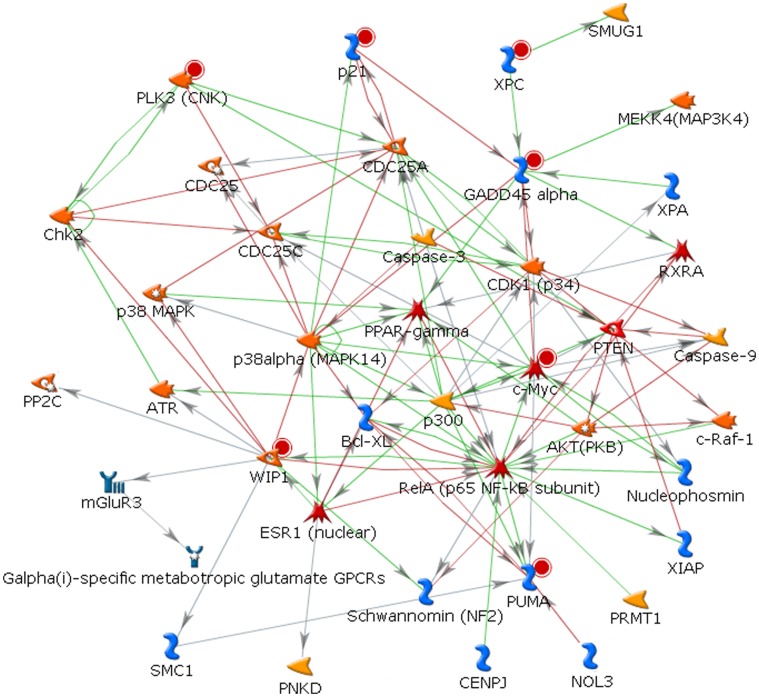
The most probable interaction network when 20 genes were entered into MetaCore software. The resulting interacting network uses only 7 genes. Red, green, and gray lines indicate inhibitory, stimulatory, and unspecified interactions, respectively.

**Table 5:** The results of the proposed scoring function test applied to the network in **Figure 5** **.**

Ranking	Protein	Gene symbol		Score		GSE1977 *p*-value	GSE23393 *p*-value	No. of edges
1	c-Myc		MYC		113.74	0.02420	0.14898	12
2	GADD45 alpha		GADD45A		110.34	8.63E-06	0.00172	9
3	WIP1		PPM1D		108.16	0.00044	0.00310	11
4	PUMA		BBC3		102.70	0.07171	0.01019	6
5	p21		CDKN1A		100.13	0.00027	0.00367	3
6	PLK3 (CNK)		PLK3		99.70	0.00285	0.08072	4
7	XPC		XPC		85.62	0.00068	0.03330	2

### Gene Ranking and Identification of a Core Radio-response Network

**Figure 5** shows the most probable/robust single interaction network when the 20 overlapping genes were entered into the MetaCore software. Of the 20 input genes, seven genes appeared in this core radio-response network. We applied our graph-based scoring function to this network and the results are summarized in **Table 5** **.** MYC was ranked first with a score of 113.74, which had a high *p*-value in GSE23393 and a statistically significant *p*-value, yet still relatively high compared to other genes, in GSE1977. As a hub gene, MYC had the highest number of edges (*n* = 12) that seem to contribute to the score. Overall *p*-values in GSE23393 (*in situ* IR) are higher than those of GSE1977 (*ex vivo* IR). Intuitively, as the number of edges increases, the score seems to increase. However, it should be noted that although GADD45A has 9 edges, it obtained a higher score than PPM1D, which has 11 edges. This is attributed to the fact that when we calculate the score for a gene, our scoring function takes into account all network interactions and the number of references on the interactions in the network. Interestingly, CDKN1A obtained a relatively high score of 100.13, considering only 3 edges and substantially low *p*-values (0.00027 in GSE1977 and 0.00367 in GSE23393).

## Discussion

We have demonstrated an unbiased bioinformatics filtering methodology to objectively identify a core network of key interacting genes that are important to radiation response. We hypothesized that, by combining several different types of datasets, we are increasingly likely to identify interacting genes that are particularly important to radiation response. We also hypothesize that these genes are therefore attractive candidates for biomarker testing. For example, the 7 key genes contain 89 relevant SNPs in our radiation therapy cancer dataset and we are in the process of testing late toxicity with the dataset. We make no claim that the network shown in **Figure 5** dominates radiation response and do not expect that to be the case. Nevertheless, this network seems to be highly relevant to radiation response: among the 7 genes, 5 and 4 genes are involved in cell cycle control and apoptosis, respectively. More detailed information is shown in **Table S2**. Five of these genes, including MYC, BBC3, GADD45A, CDKN1A, and XPC belong to a list of 34 radio-responsive genes observed by Tusher *et al*. [187]. Moreover, this network is consistent with (though slightly different from) the programmed cell death network reported by Moussay *et al.* [189].

**Figure 4** shows the number of genes commonly or uniquely identified among three different studies (literature review and analysis of two microarray datasets). Interestingly, relatively few genes overlapped among the three analyses. Literature coverage is expected to be incomplete regarding coverage of radiosensitivity genes. Microarray analysis is subject to high false positive and false negative rates [190]. Another possible reason for the small number of overlapped genes is the widely differing irradiation conditions and doses. Despite this, the biological processes and pathways generated from the 20 overlapping genes were similar to those generated from the whole literature review.

We further analyzed the 20 genes, uploading these genes into the MetaCore software. In the network of the most probable biological process shown in **Figure 5**, only seven out of 20 genes appeared in the network. Additional genes were automatically added to the network by MetaCore, including AKT1, RELA, BCL2L1, PTEN, CDK1, and XIAP. Note, however, that these genes were also members of the list generated by our radiation response literature review, suggesting some consistency between these sources. This also suggests a potential ability to find novel biomarker candidates through the network mapping/ranking process, though that did not occur in this case.

The graph-based scoring function proposed in our previous study [7] was modified and applied to the network shown in **Figure 5**. In some studies, researchers tend to regard genes with high degrees of connectivity (hub genes) as significant in an interaction network, while neglecting others [4]. While this is rational, finding hub genes based on edge connectivity considers only direct interactions between genes whereas our proposed approach takes into account all interactions in a network (that is, the entire graph structure) and the number of published references on the interactions. To measure the closeness between two proteins (say *A* and *B*), we employed two scores; a node score and a reference score. In a protein interaction network, it is obvious that as the number of internal nodes between *A* and *B* increases, these two proteins are less likely to be related with each other. In contrast, the reference score is a score calculated using the number of papers that studied on an interaction between two proteins, which can be important evidence that there is an actual relationship between the two proteins. As can be seen in **Table S3**, MYC was first ranked using a total score. However, BBC3 and PPM1D were first ranked using a reference score and a node score, respectively. CDKN1A, PLK3, and XPC obtained somewhat high scores considering their connectivity, suggesting that they could play important roles in this core network. We believe that the use of both scores could be more effective for ranking proteins in a protein interaction network.

Future work will test SNPs identified in this network against toxicity resulting from radiation therapy. As the number of patients available for SNP analyses increases, it may be rational to expand the number of candidate SNPs to several hundreds or more. The general methodology may be applied in many genetic/protein biomarker studies with limited patient data.

## Supporting Information

Figure S1 **A normal quantile plot of t-scores for GSE23393 after 10,000 permutations.**
(TIF)

Figure S2 **Significant gene detection.** A volcano plot that depicts the –log10 of *q*-values against log2 of fold changes for all genes in GSE23393.
(TIF)

Table S1 The top ten GeneGo pathways/processes and GO processes generated by the MetaCore software when 20 overlapped genes were used.
(DOC)

Table S2 Biological processes for the seven genes shown in **Table 5**.
(DOC)

Table S3 Scores obtained using the graph-based scoring function.
(DOC)
