# Supplementary figures and images for: A Bioinformatics Filtering Strategy for Identifying Radiation Response Biomarker Candidates
Source: PLoS One. 2012 Jun 29;7(6):e38870. doi: 10.1371/journal.pone.0038870 (PMC3387230; doi:10.1371/journal.pone.0038870)

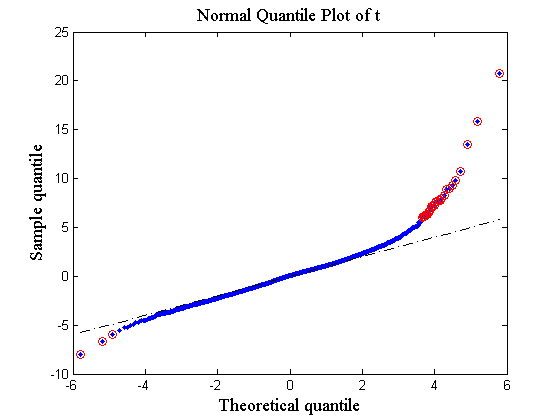

Supplement: Figure S1 — A normal quantile plot of t-scores for GSE23393 after 10,000 permutations. (TIF) [file pone.0038870.s001.tif]

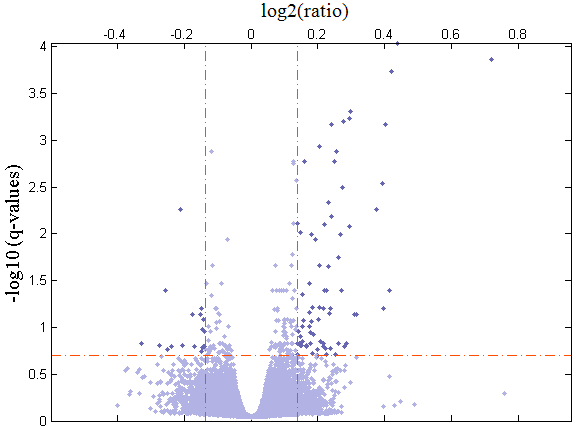

Supplement: Figure S2 — Significant gene detection. A volcano plot that depicts the –log10 of q-values against log2 of fold changes for all genes in GSE23393. (TIF) [file pone.0038870.s002.tif]
