# Supplementary material for: A Bioinformatics Filtering Strategy for Identifying Radiation Response Biomarker Candidates
Source: PLoS One. 2012 Jun 29;7(6):e38870. doi: 10.1371/journal.pone.0038870 (PMC3387230; doi:10.1371/journal.pone.0038870)
Supplement: Table S1 — The top ten GeneGo pathways/processes and GO processes generated by the MetaCore software when 20 overlapped genes were used. (DOC) [file pone.0038870.s003.doc]

**Table S1.** The top ten GeneGo pathways/processes and GO processes generated by the MetaCore software when 20 overlapped genes were used.

| Ranking | GeneGo Pathways |
| --- | --- |
| 1  2  3  4  5  6  7  8  9  10 | DNA damage_Brca1 as a transcription regulator  DNA damage_ATM/ATR regulation of G1/S checkpoint  Signal transduction_AKT signaling  Apoptosis and survival_Apoptotic TNF-family pathways  DNA damage_ATM / ATR regulation of G2 / M checkpoint  Apoptosis and survival_p53-dependent apoptosis  DNA damage_Role of Brca1 and Brca2 in DNA repair  DNA damage_Nucleotide excision repair  Transcription_P53 signaling pathway  Cytoskeleton remodeling_TGF, WNT and cytoskeletal remodeling |
| Ranking | GeneGo Processes |
| 1  2  3  4  5  6  7  8  9  10 | DNA damage_Checkpoint  Cell cycle_G1-S  Proliferation_Negative regulation of cell proliferation  Cell cycle_G2-M  Apoptosis_Death Domain receptors & caspases in apoptosis  Apoptosis_Apoptosis stimulation by external signals  Development_Skeletal muscle development  DNA damage_BER-NER repair  Inflammation_IL-6 signaling  Reproduction_Male sex differentiation |
| Ranking | GO Processes |
| 1  2  3  4  5  6  7  8  9  10 | G2/M transition of mitotic cell cycle  Response to DNA damage stimulus  Cellular response to stress  Response to abiotic stimulus  Vascular smooth muscle contraction  Regulation of apoptosis  Regulation of programmed cell death  Regulation of cell death  Interphase of mitotic cell cycle  Interphase |
