# Supplementary material for: A Bioinformatics Filtering Strategy for Identifying Radiation Response Biomarker Candidates
Source: PLoS One. 2012 Jun 29;7(6):e38870. doi: 10.1371/journal.pone.0038870 (PMC3387230; doi:10.1371/journal.pone.0038870)
Supplement: Table S2 — Biological processes for the seven genes shown in Table 5 . (DOC) [file pone.0038870.s004.doc]

**Table S2.** Biological processes for the seven genes shown in **Table 5**.

|  | DNA repair | Cell proliferation | Cell cycle | Apoptosis | Response to stress |
| --- | --- | --- | --- | --- | --- |
| MYC |  | **v** | **v** | **v** |  |
| GADD45A | **v** |  | **v** | **v** | **v** |
| PPM1D |  | **v** | **v** |  |  |
| BBC3 |  |  |  | **v** |  |
| CDKN1A |  | **v** | **v** | **v** | **v** |
| PLK3 |  |  |  |  |  |
| XPC | **v** |  | **v** |  | **v** |
