# Supplementary material for: A Bioinformatics Filtering Strategy for Identifying Radiation Response Biomarker Candidates
Source: PLoS One. 2012 Jun 29;7(6):e38870. doi: 10.1371/journal.pone.0038870 (PMC3387230; doi:10.1371/journal.pone.0038870)
Supplement: Table S3 — Scores obtained using the graph-based scoring function. (DOC) [file pone.0038870.s005.doc]

**Table S3.** Scores obtained using the graph-based scoring function.

| Ranking | Protein | Gene symbol | Total score | Reference score | Node score |
| --- | --- | --- | --- | --- | --- |
| 1 | c-Myc | MYC | 113.74 | 34.05 | 85.28 |
| 2 | GADD45 alpha | GADD45A | 110.34 | 33.43 | 81.84 |
| 3 | WIP1 | PPM1D | 108.16 | 28.07 | 87.30 |
| 4 | PUMA | BBC3 | 102.70 | 38.89 | 75.52 |
| 5 | p21 | CDKN1A | 100.13 | 35.43 | 69.85 |
| 6 | PLK3 (CNK) | PLK3 | 99.70 | 37.18 | 68.03 |
| 7 | XPC | XPC | 85.62 | 25.01 | 60.61 |
